# Primary giant cell tumour of the breast with recurrence: A rare case report

**DOI:** 10.4102/sajr.v26i1.2393

**Published:** 2022-04-20

**Authors:** Farhana E. Suleman, Moipone N. Vilakazi, Meshack Bida, Richard Edwards

**Affiliations:** 1 Department of Radiology, Faculty of Health Sciences, University of Pretoria, Pretoria, South Africa; 2 Department of Anatomical Pathology, Faculty of Health Sciences, University of Pretoria, Pretoria, South Africa

**Keywords:** giant cell tumour, breast tumour, GCT-ST, breast imaging

## Abstract

Giant cell tumour (GCT) arising from the soft tissues of the breast is a rare disease with only eight cases previously reported in the literature. We present a case of histologically proven GCT of the breast, which demonstrated recurrence a few months after resection.

## Introduction

Microscopically, giant cell tumours (GCTs) are diagnosed when an even distribution of multinucleated osteoclast-like giant cells (OGCs) are noted surrounded by oval and spindle mononuclear cells.^1^ They usually occur in the epiphysis of skeletally mature long bones and are called GCT of the soft tissues (GCT-ST) when they occur in the soft tissue instead of bone.^1^ Giant cell tumours of the soft tissues are usually found in the superficial and deep soft tissues of the extremities but have been described in the pancreas, lung, thyroid gland, urothelial tract, skin, larynx, heart and very rarely, in the breast.^1,2^

Breast carcinomas containing OGCs compromise less than 2% of all breast cancer cases.^3^ However, primary GCT of the breast resembling the one found in bone, and not related to underlying breast carcinoma is extremely rare with only eight other cases found, described in the literature between 1981 and 2020.^1,2,4,5,6,7,8,9^

This report focuses on a 58-year-old woman with histologically proven primary OGC-ST of the breast, with a discussion on the radiological and histological findings, and a review of the available literature.

## Case report

A 58-year-old woman was referred with a self-detected mass in the left breast. She had no past or family history of breast disease and no underlying comorbidities. Clinically on palpation, a firm, non-tender, well-defined left-sided breast mass measuring approximately 210 mm × 160 mm was noted extending into the axilla. Ipsilateral axillary lymphadenopathy was palpated with no contralateral, supraclavicular or infraclavicular lymphadenopathy present. The clinical diagnosis of a phyllodes tumour was made, and the patient underwent a core biopsy of the mass and ipsilateral axillary lymph node by the surgeon prior to imaging.

**FIGURE 1:**
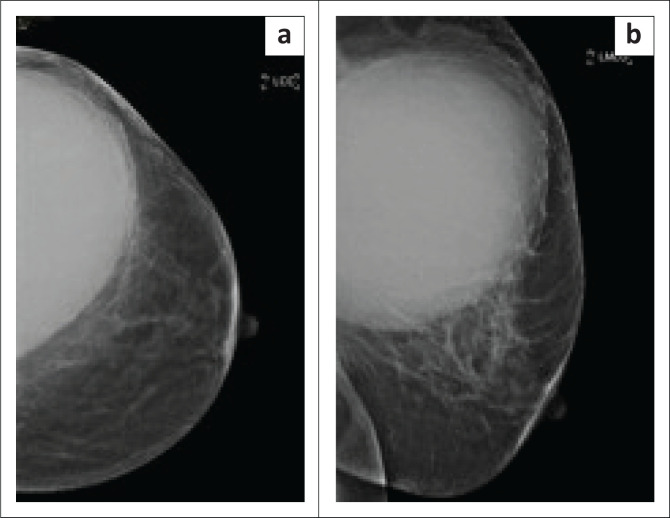
Cranio-caudal view of the left breast on initial mammogram (a) and mediolateral oblique view (b) demonstrating a well-defined, hyper-dense mass within the supero-lateral aspect of the breast.

**FIGURE 2:**
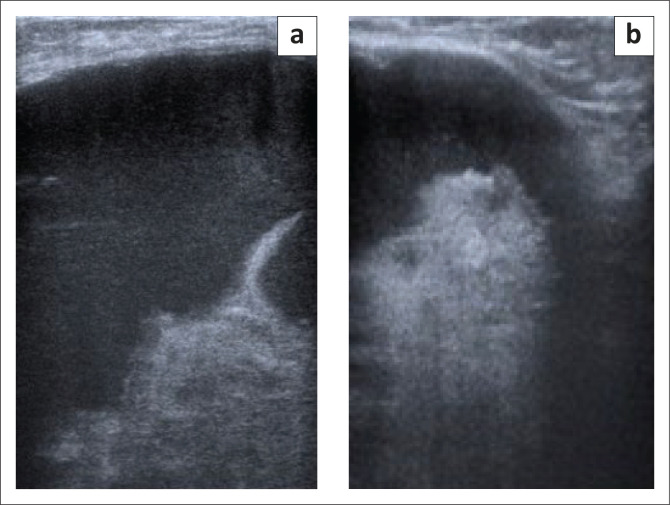
Axial/transverse view (a) and sagittal/ longitudinal view (b) of the left breast on initial ultrasound, demonstrating a large anechoic mass with multiple septations and irregular walls with debris.

She was then referred to the radiology department for a mammogram and a breast ultrasound. The left mammogram demonstrated a 126 mm × 110 mm, circumscribed, round mass within the upper outer quadrant. The mass was homogenous with mass effect on the adjacent glandular tissue as displayed in Figures 1a and 1b. Ultrasound of the left breast demonstrated a large anechoic mass with septations and an irregular inner thickened wall with debris as displayed in Figures 2a and 2b. The right mammogram and ultrasound were within normal limits. The mass was classified as a Breast Imaging-Reporting and Data Systems (BI-RADS) 4A lesion and the patient was referred back to the surgeons. An image-guided biopsy was not performed at this stage because the patient was awaiting histology results for the biopsy performed by the surgeon.

Subsequent biopsy results of the mass and lymph node showed features compatible with organising fat necrosis with a reactive lymph node. This was thought to be incompatible with the clinical imaging findings and therefore not representative of the pathology. Unfortunately, because of the long waiting times for MRI and the size of the lesion, the decision was made to take the patient for surgery. The patient then underwent a left-sided mastectomy; her tumour node metastases (TNM) staging prior to surgery was T3N0Mx.

**FIGURE 3:**
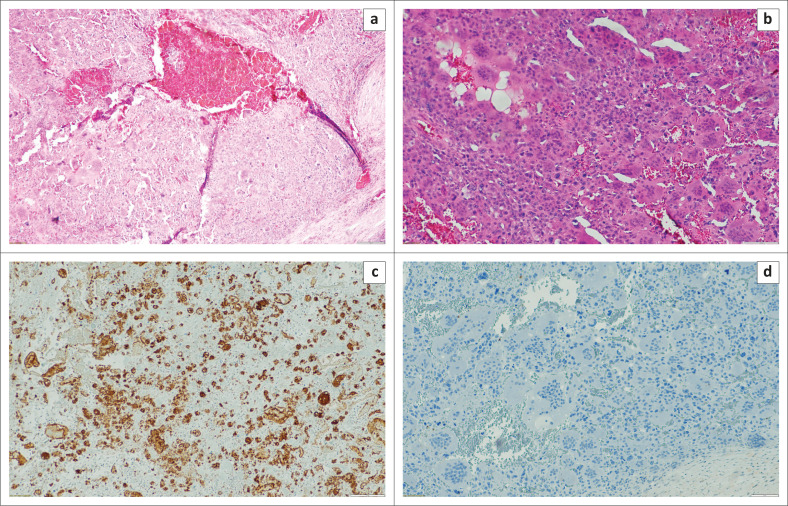
Histology of the surgical specimen. The tumour was composed mainly of oval and spindle mononuclear histiocyte-like cells and multinucleated giant cells; hematoxylin and eosin stain, × 4 (a) × 20 (b). Immunohistochemistry of the tumour. Oval and spindle mononuclear histiocyte – like cells and multinucleated giant cells stain positively for CD68 (c) and negatively for AE1/3 and MNF – 116 (d).

Histology of the mastectomy specimen revealed a large well-encapsulated tumour with necrotic debris. The tumour was multinodular and consisted of sheet-like arrangements of neoplastic mononuclear cells with evenly interspersed multinucleated OGCs. These mononuclear cells appeared plump, resembling histiocytic cells, admixed with oval to spindle-shaped cells, resembling myofibroblasts as demonstrated in Figures 3a and 3b. There was no carcinomatous component present in all the sections examined.

Nuclear atypia, characterised by nuclear enlargement, hyperchromasia and pleomorphism, was noted with as many as four mitotic figures per 10 high power field in some areas. In addition, aneurysmal bone cyst-like spaces were focally seen, containing red blood cells, but not lined by endothelium.

CD68 stained the OGCs as well as some of the intervening histiocytic cells. MNF-116 and AE1/3 were negatively immunoreactive (Figures 3c, 3d). The results were in keeping with a GCT arising from the soft tissues of the breast.

**FIGURE 4:**
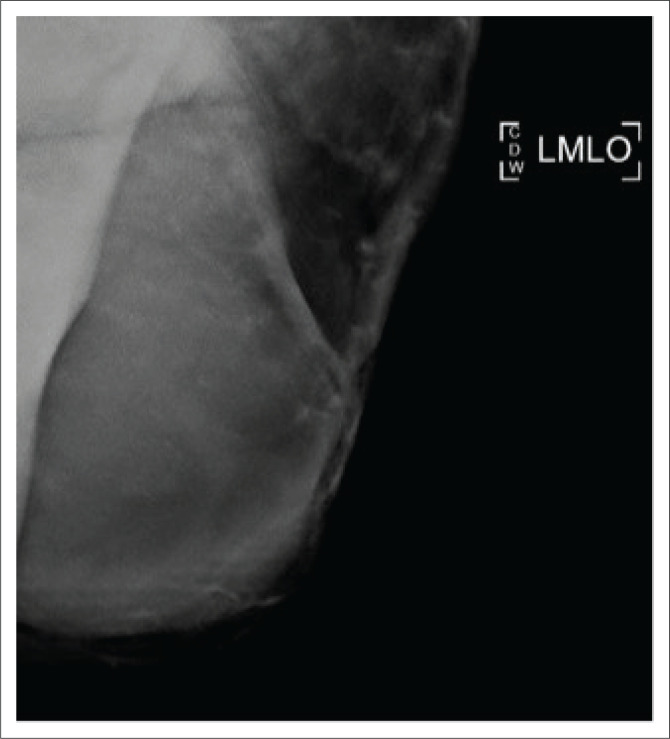
Post-surgical, medio-lateral oblique view of the left mastectomy scar demonstrating a hyper-dense mass within the axillary tail.

**FIGURE 5:**
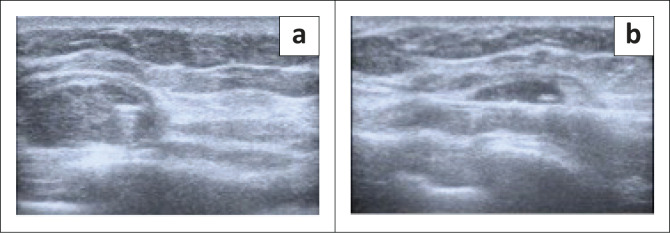
Post-surgical, axial (a) and sagittal (b) views of the left breast on ultrasound demonstrating the largest hypoechoic lesions at the inferior-lateral aspect of the scar. This lesion was not separable from the underlying pectoralis muscle.

The patient then defaulted on her follow-up appointments including an appointment for a staging CT scan. Five months after the mastectomy, she was referred for a repeat mammogram and ultrasound for multiple large lesions at the left mastectomy site. The mammogram of the left scar demonstrated a 50 mm × 60 mm hyper-dense mass within the axillary tail as displayed in Figure 4. Ultrasound of the left mastectomy site demonstrated round hypoechoic lesions at the scar as demonstrated in Figures 5a and 5b. The largest measured 20 mm × 11 mm, situated at the inferior-lateral aspect of the scar and not separable from the underlying pectoralis muscle. Large axillary masses consisting of fluid with areas in keeping with soft tissue densities were demonstrated; the largest measured 56 mm × 38 mm. The lesion was classified as BI-RADS 4C. Ultrasound-guided core biopsy of the largest lesion demonstrated a poorly differentiated malignancy with OGCs – features in keeping with recurrence. The patient underwent a single cycle of radiotherapy and was then referred to oncology where she was treated with adriamycin and continues follow-up.

## Discussion

Commonly GCT-ST is found in the superficial and deep soft tissues of the extremities but has been described in the pancreas, lung, thyroid gland, urothelial tract, skin, larynx, heart and very rarely in the breast.^1,2^ Osteoclast-like giant cell tumour of the soft tissues is histologically and immunophenotypically similar to GCT arising in the epiphysis of long bones with a diffuse distribution of OGCs, but it is important to evaluate the background component of mononuclear cells to make the diagnosis.^5^

Osteoclast-like giant cell tumour of the soft tissues of the breast is a rarely diagnosed primary tumour of the breast with only eight cases previously reported in the literature.^1,2,4,5,6,7,8,9^ The prognosis is therefore still unknown. Although it is usually considered a benign tumour, it may recur locally but seldom metastasizes.^5^ There are reports, however, describing osteoclast-like giant cell tumour of the soft tissue (OGCT-ST) as a biologically heterogeneous group of tumours ranging from benign to highly malignant.^1^ Because it is so rare the prognosis is uncertain and standard therapy has not yet been established.^8^

This case is the only OGT-ST of the breast to report local recurrence with aggressive behaviour. Pulmonary metastases and death were reported in another case of OGCT of the breast.^5^ No associated breast carcinoma was noted in either case. Clinically the majority of these patients have presented with large masses, with the current case being the second to be clinically assessed as a phyllodes tumour.^6^ One reported case was associated with an intraductal papilloma^7^ and another had an adjacent separate tumour, histologically in keeping with ductal carcinoma *in situ*.^9^ On imaging, the majority of the cases reported well circumscribed tumours on mammogram, and all had hypoechoic cystic regions with solid tumour on ultrasound. All the gross specimens reported necrosis.

The differential diagnosis to be considered in this case would include a multitude of other carcinomas, including breast cancer with OGCs and other tumours presenting with abundant giant cells such as leiomyosarcoma, osteosarcoma, malignant fibrous histiocytomas and metastatic GCT of bone.^5,7^ Underlying carcinoma of the breast with OGC’s is also uncommon and occurs in less than 2% of breast carcinoma cases.^3^ While these tumours are most commonly moderately or poorly differentiated invasive ductal carcinoma,^10^ a rare subtype of metaplastic carcinoma with osteoclast-like giant cells, comprising 11% of metaplastic carcinoma,^11^ should be differentiated from primary GCT of the soft tissues of the breast.^12^ A lack of epithelial component, marked cellular atypia, and pleomorphism differentiates GCT from these tumours.

Pathological assessment of the surgical specimen from the patient presented, demonstrated a relatively homogeneous and bland-appearing feature and only mild degree of nuclear pleomorphism throughout the tumour, which excluded breast cancer with OGCs, leiomyosarcoma, osteosarcoma, and malignant fibrous histiocytoma. Metastatic GCT of the bone was excluded by the absence of a history of GCT of the bone.

## Conclusion

Giant cell tumour of the soft tissues of the breast is an extremely rare tumour with the potential to recur locally and to metastasize. Long-term prognosis is therefore uncertain and the importance of long-term clinical and radiological follow-up after resection is emphasised in order to detect any form of possible recurrence early and ensure swift, concise treatment options.
